# Acute distal tibiofibular syndesmosis injury: a systematic review of suture-button versus syndesmotic screw repair

**DOI:** 10.1007/s00264-012-1500-2

**Published:** 2012-02-09

**Authors:** Tim Schepers

**Affiliations:** Department of Surgery-Traumatology, Erasmus MC, University Medical Center Rotterdam, Room H822-k, P.O. Box 2040, 3000 CA Rotterdam, The Netherlands

## Abstract

**Purpose:**

Recently, a new suture-button fixation device has emerged for the treatment of acute distal tibiofibular syndesmotic injuries and its use is rapidly increasing. The current systematic review was undertaken to compare the biomechanical properties, functional outcome, need for implant removal, and the complication rate of syndesmotic disruptions treated with a suture-button device with the current 'gold standard', i.e. the syndesmotic screw.

**Method:**

A literature search in the electronic databases of the Cochrane Library, EMbase, Pubmed Medline, and Google Scholar, between January 1st 2000 to December 1st 2011, was conducted to identify studies in which unstable ankle fractures with concomitant distal tibiofibular syndesmotic injury were treated with either a syndesmotic screw or a suture-button device.

**Results:**

A total of six biomechanical studies, seven clinical full-text studies and four abstracts on the TightRope system, and 27 studies on syndesmotic screw or bolt fixation were identified. The AOFAS of 133 patients treated with TightRope was 89.1 points, with an average study follow-up of 19 months. The AOFAS score in studies with 253 patients treated with syndesmotic screws (metallic and absorbable) or bolts was 86.3 points, with an average study follow-up of 42 months. Two studies reported an earlier return to work in the TightRope group. Implant removal was reported in 22 (10%) of 220 patients treated with a TightRope (range, 0–25%), in the screw or bolt group the average was 51.9% of 866 patients (range, 5.8–100%).

**Conclusion:**

The TightRope system has a similar outcome compared with the syndesmotic screw or bolt fixation, but might lead to a quicker return to work. The rate of implant removal is lower than in the syndesmotic screw group. There is currently insufficient evidence on the long-term effects of the TightRope and more uniform outcome reporting is desirable. In addition, there is a need for studies on cost-effectiveness of the treatment of acute distal tibiofibular syndesmotic disruption treated with a suture-button device.

## Introduction

It is estimated that 10% of all ankle fractures and 20% of operatively treated ankle fractures are accompanied by a syndesmotic injury [12, 16, 49, 54, 62]. The quest for the best treatment of acute distal tibiofibular syndesmotic disruption is still in full progress. Because of the contradicting goals in treatment, i.e. rigid fixation for an adequate period on one hand versus early return to full range of motion at the syndesmosis on the other, various different strategies have been used throughout the years. Among these are the metallic syndesmotic screw, which is currently considered the 'gold standard', bioabsorbable screws, bolt-fixation, syndesmotic hook, integrated syndesmotic fixation with nail (ANK), staples, direct repair, and the use of suture-loops with or without endobuttons [15, 26, 36, 40, 47, 56]. Of the latter devices the suture-button device is increasingly popular [59]. The use of a suture (with or without buttons) to support the ruptured syndesmotic ligaments is not new, but its use has increased since the introduction of a pre-assembled suture-button device (TightRope® syndesmotic repair kit; Arthrex, Inc., Naples, FL). This system is compiled of two No5 braided polyester sutures and two titanium or stainless steel endobuttons.

The theoretical advantages of a suture-button device over a metallic syndesmotic screw are that it allows physiologic motion at the syndesmosis while maintaining the reduction, less risk of hardware pain and subsequent implant removal, and it permits earlier return to motion as there is no risk of screw breakage and subsequent recurrent syndesmotic diastasis.

The current systematic review was primarily undertaken to gain insight in the overall biomechanical properties, functional outcome, need for implant removal, and the complication rate of unstable ankle fractures with concomitant syndesmotic injury treated with a suture-button device. The second aim was to determine how these results compare to the current 'gold standard' (i.e. the syndesmotic screw).

## Material and methods

### Literature search

A literature search was conducted to identify studies in which unstable ankle fractures with concomitant distal tibiofibular syndesmotic injury were treated with either a syndesmotic screw or a suture-button device. The electronic databases up to December 1st 2011 of 'the Cochrane Library', 'Pubmed Medline', 'EMbase', and 'Google Scholar' were explored using the combination of the following search-terms and Boolean operators: syndesmo* OR tibiofibular AND ankle OR distal fibula AND tightrope OR suture button OR screw. No restriction in language and publication date was applied. Publications were requested at the university medical (internet) library and reviewed. In addition, a comprehensive search of reference lists of all identified articles was conducted to find additional studies. An article was found eligible when it concerned (1) the treatment of an acute syndesmotic disruption or (2) use of a suture-button device or a syndesmotic positioning screw (metallic or absorbable) as a surgical technique. Abstracts from scientific meetings were included in the current review when sufficient data could be extracted on functional outcome or complication rate.

The biomechanical properties, functional outcome, need for implant removal and complication rate of the suture-button system was compared with the results of acute syndesmotic injuries treated with a metallic or bioabsorbable syndesmotic screw or comparable implant. These studies were identified using a similar search strategy. Because of the contemporary use of suture-button devices, only studies considering the use of a syndesmotic screw from 2000 to 2011 were included to provide insight into current practice, and to make the results more comparable.

## Results

A total of two studies were excluded beforehand as being solely technical descriptive manuscripts [8, 57]. One study was excluded being a radiological awareness review [41]. The number of studies available is shown per section: biomechanical and functional outcome.

### Biomechanical

A total of six biomechanical studies were identified using a suture-button device [17, 29, 49, 52, 55, 58]. The study by Miller et al. [36] was not included, as a suture (no. 5 braided polyester) without buttons looped through drill holes at two levels in the tibia and fibula for the fixation of a syndesmotic disruption was used.

An overview of the six included biomechanical studies using a suture-button device and key findings is shown in Table 1. Below is a summary of the different studies and testing protocols.

**Table 1:** Biomechanical studies of suture-button repair of distal tibiofibular syndesmotic injuries

Study	Intervention	Control	Main study conclusions
Seitz et al. (1991) [49]	10 FFCA with No5 braided polyester suture and polyethylene buttons	10 FFCA with single 3.5 mm tri-cortical screw	Pull-out strength SB lower, but more consistent. Less dependent on bone quality. Failure always through button
Thornes et al. (2003) [58]	8 ECA with No5 braided polyester suture and metallic endobuttons	8 ECA with single 4.5 mm four-cortical screw	No significant difference between SB and screw fixation. SB more consistent performance
Forsythe et al. (2008) [17]	10 FFCA with TightRope	10 FFCA with single 4.5 mm four-cortical screw	Significantly greater diastasis in the suture-button group at all external rotation loads. No hardware failures. Screw failed at lower load compared to the suture-button
Soin et al. (2009) [52]	10 FFCA with two TightRopes	10 FFCA with single 3.5 mm four-cortical screw	No significant difference in translation and rotation between SB and screw. Screw had significantly greater failure torque versus SB. Two SB behave similarly to the syndesmotic screw in the syndesmotic rupture injury model
Klitzman et al. (2010) [29]	8 FFCA with TightRope	Same 8 FFCA with single 3.5 mm screw	Syndesmotic gap after testing not significantly different between intact and the SB group, screw group had significantly smaller gap
Teramoto et al. (2011) [55]	6 FFCA sequentially tested intact, syndesmotic injury, single TightRope, double TightRope, anatomical TightRope, and 3.5-mm screw model		Screw most rigid fixation, anatomical SB adequate fixation, single and double SB insufficient stabilization in multidirectional testing

*FFCA* fresh frozen cadaver ankles, *ECA* embalmed cadaver ankles, *SB* suture-button device

In the study by Seitz et al. [49], 20 cadaver legs (level of amputation unknown) were used. The talus was disarticulated from the ankle joint, and all surrounding soft tissues were removed. All syndesmotic ligaments were divided and a pull-out (pull-apart) test to failure was performed comparing a self-constructed suture-button device made of a double No5 braided polyester suture and polyethylene buttons.

Thornes et al. [58] used 16 embalmed cadaver legs (level of amputation unknown) of which the medial deltoid and syndesmotic ligaments were sectioned. The testing included 12.5 Nm rotation stress comparing the suture-button device using metallic buttons and a 4.5-mm screw. The principal author is designer of the currently used suture-button device and patented the device [58].

Forsythe et al. [17] used ten cadavers amputated above the knee, which were tested using 12.5-Nm external rotation after sectioning of the deltoid ligament, distal 15 cm of the interosseous membrane, and anterior tibiofibular ligament. The posterior tibiofibular ligament and the fibula were left intact (Boden model). The TightRope system and a 4.5-mm screw were compared. Worth mentioning is that this study was funded by the manufacturer of the suture-button device.

Soin et al. [52] used 20 cadaver legs disarticulated at knee level, and divided the anterior tibiofibular, posterior tibiofibular, deltoid, and interosseous ligaments using minimal soft tissue dissection. The testing protocol used cyclic axial compression with 750 N, 7.5-Nm external rotation, and a combination of both, while comparing a 3.5-mm screw and two TightRopes.

Klitzman et al. [29] used eight cadaver legs amputated below the knee, and with minimal soft tissue dissection the anterior, posterior, transverse, interosseous tibiofibular, and deltoid ligaments were sectioned. With an axial load of 50 N and 5 Nm torque, the TightRope system and a 3.5-mm screw were compared to investigate the syndesmotic diastasis after cycling at submaximal loads, laxity due to cycling, and fibular movement in the sagittal plane.

Teramoto et al. [55] used six above-knee amputated cadaver legs. The similar syndesmotic disruption model was used as Forsythe, with the anterior tibiofibular ligament, the distal 15 cm of the interosseous membrane, and the deltoid ligament divided. Using 5-Nm external rotation (in dorsiflexion and inversion) an intact syndesmosis, a syndesmotic injury model, single TightRope fixation, double TightRope, anatomical TightRope placement, and 3.5-screw model were sequentially tested.

### Functional outcome

Two studies were not included in the functional outcome analysis. The study by Seitz et al. [49] was one, because it used a device, which was not pre-assembled, with polyethylene buttons which appeared to be the weakest link in testing. Newer devices used metallic endobuttons. In the clinical part of the study by Seitz et al., 11 of the 12 Weber-C injuries regained pre-injury levels at an average follow-up of 3.2 years, there were no device failures, and all were routinely removed after eight to 12 months [49]. The other excluded study was by Nelson [40]; who used three strands of no. 2 nonabsorbable sutures looped around a fibular and tibial screw.

Four abstracts following a scientific meeting, published in well renowned journals, were identified and were found adequately usable [18, 34, 44, 60].

A total of eight full-text studies were identified, of which one used the same data considering the patients with a TightRope but added a control group (with a syndesmotic screw) in the second publication [9–11, 14, 39, 42, 59, 64]. Only the latter was used in the current analysis.

The cardinal study characteristics and key results of the seven full-text and four abstracts are shown in Table 2. Below is a summarized description per included full-text article.

**Table 2:** Study characteristics and key results

Study	Patients (*n*)	Control (n)	LOE	Follow-up (months)	Score (max)	Points (P vs C)	Implant removal	Complications	Implant failure
Thornes et al. (2005) [59]	16	16	3	12	AOFAS (100)	93 vs 83	0 vs 12	None	None
					SB earlier return to work				
McMurray et al. (2007) [34]^a^	16	None	4	5	AOFAS	87	2	1	None
Cottom et al. (2008–2009) [11]	25	25	3	10	modAOFAS (63)	51 vs 54	0 vs 17	N.A.	None
					SF12	102 vs 102			
Coetzee and Ebeling (2009) [9]	12	12	1	28	AOFAS (100)	94 vs 88	1 vs 1	N.A.	None
Gadd et al. (2009) [18]^a^	38	None	4	14–42	N.A.	N.A.	3	2	None
Rajkumar et al. (2009) [44]^a^	12	12	3	14	OMAS (100)	86	N.A.	N.A.	N.A.
					SB earlier mobilization and return to work				
Treon et al. (2009) [60]^a^	18	None	4	4–41	N.A.	N.A.	4	6	2
Willmoth et al. (2009) [64]	6	None	4	5	N.A.	N.A.	2	None	None
DeGroot et al. (2011) [14]	24	None	4	20	AOFAS (100)	94	6	None	None
Qamar et al. (2011) [42]	16	None	4	26	AOFAS (100)	86.9	1	2	None
Naqvi et al. (2011) [39]	49	None	4	24	AOFAS (100)	85.6	3^b^	2	None
					FADI (100)	81.2			

*P* patient (suture-button), *C* control (syndesmotic screw), *SB* suture-button device, *AOFAS* American Orthopaedic Foot Ankle Society, *OMAS* Olerud Molander Ankle Score, *FADI* Foot/Ankle Disability Index, *N.A.* not available

^a^ Abstract at scientific meeting

^b^ Removals prior to technical alteration

Thornes et al. performed a non-randomized prospective trial in which patients treated by the leading author received a suture-button device and in his absence patients were treated with a syndesmotic screw by others from the same institution [59]. The fractures were classified as Weber-C in all cases, and all patient characteristics were comparable in both groups. The conflict of interest statement reported funding from a patent concerning the suture-button device.

Cottom et al. published their series treated with a TightRope in 2008. In 2009 apparently the outcome of these same patients was compared to a series of patients treated with a syndesmotic screw [10, 11]. The study type is not stated in both articles, but is most likely a retrospective comparative because of the difference in follow-up. There were eight Weber-B, five Weber-C, four Maisonneuve, and eight pure ligamentary injuries in the TightRope group. A modified AOFAS-score was used without the physical exam components, and a maximum of 63 points.

Willmott et al. showed, in a series of six patients (four Weber-C, one Maisonneuve, one ligamentary diastasis), a previously unreported high incidence of implant removal [64]. A total of two TightRopes needed removal due to wound irritation (granuloma formation).

Coetzee et al. published an interim analysis of their randomized trial in which 12 patients in both study arms were included [9]. It is unclear which fracture types were included in both groups. A non-significant improvement in range of motion, mainly plantar-flexion, was reported (*p* = 0,054). The study is possibly still ongoing and hopefully the Olerud Molander Ankle Score will be included at the final follow-up to make a better comparison with the available literature.

DeGroot et al. reported on 24 patients in a retrospective fashion [14]. Fractures were classified according to the Lauge-Hansen classification. In most patients two Tight-Ropes were placed. This study was the first to report osteolysis and subsidence of the suture button through the cortex of the fibula or tibia and slight enlargement of the tibial tunnel, which appeared to more likely occur with longer follow-up.

Qamar et al. reported the results of 16 patients with predominantly Weber-C injuries [42]. The single indication where the implant was removed was probably due to the sutures being cut off too short. Even though the authors do not report on the subsidence of the suture button through the cortex, their radiographic images show this effect at follow-up after 24 months.

The study by Naqvi et al. is the largest series thus far [39]. A total of six Weber-B, 29 Weber-C, 11 Maisonneuve, and three ligamentary injuries were included. At a certain point in time the authors modified their technique to bury the knot at the lateral side. Using this modification no implant removed has been needed since.

### Outcome comparison with the ‘gold standard’

The main difficulty in comparing the functional outcome between the syndesmotic screw and the TightRope system is that most studies using the positioning screw use the Olerud Molander Ankle Score (OMAS) (11 out of 27) and less frequently the American Orthopaedic Foot Ankle Society (AOFAS) Score (six out of 27), whereas the most frequently used functional score in the TightRope studies is the AOFAS (seven out of 11) (Tables 2 and 3).

**Table 3:** Outcome comparison

Study (year)	Patients	Follow-up (months)	Implant removal	Score (max)	Points
Kennedy et al. (2000) [28]	26	35	26	Baird-Jackson (100)	62.8
Thordarson et al. (2001) [56]	32 (17 abs)	11	15	N.A.	N.A.
Heim et al. (2002) [20]	17	12	17	N.A.	94% GE
Hovis et al. (2002) [24]	23 (abs)	34	0	OMAS (100)	94
Sinisaari et al. (2002) [51]	30 (18 abs)	20	12	OMAS (100)	85.2
Hoiness and Stromsoe (2004) [23]	64	12	32	OMAS (100)	88.9
Sproule et al. (2004) [53]	14	25	13	GFA (100)	95.6
				Shoe comfort (100)	81.7
Kaukonen et al. (2005) [27]	38 (20 abs)	35	18	N.A.	N.A.
Kukreti et al. (2005) [30]	36	35	33	N.A.	86%satisfied
Thornes et al. (2005) [59]	16	12	12	AOFAS (100)	83
Weening and Bhandari (2005) [62]	51	18	30	OMAS (100)	74.1
				SMFA (0)	11.4
Bell and Wong (2006) [5]	30	15	23	Baird-Jackson (100)	87.5
Moore et al. (2006) [38]	120	5	7	N.A.	N.A.
Rao et al. (2008) [45]	17	12	6	OMAS (100)	87.3
Ahmad et al. (2009) [1]	70 (abs)	33	2	AOFAS (100)	90 (82.8% GE)
Coetzee and Ebeling (2009) [9]	12	28	1	AOFAS (100)	88
Cottom et al. (2009) [11]	25	10	17	modAOFAS(63)	54
De Vil et al. (2009) [13]	28 (bolt)	66	5	AOFAS (100)	86
Hamid et al. (2009) [19]	52	30	27	N.A.	N.A.
Rajkumar et al. (2009) [44]	12	14	N.A.	OMAS (100)	86
Rao et al. (2009) [46]	21	12	15	OMAS (100)	81.1
Egol et al. (2010) [16]	79	12	11	AOFAS (100)	83.5
				SMFA (0)	14.5
Manjoo et al. (2010) [32]	76	23	12	LEM (100)	81.4
				OMAS (100)	60.0
Miller et al. (2010) [35]	25	3	25	OMAS (100)	75.0
Mohammed et al. (2010) [37]	12	13	12	OMAS (100)	75
Wikerøy et al. (2010) [63]	48	101	33	OMAS (100)	82.5
				AOFAS (OTA) (100)	86.5
Hsu et al. (2011) [25]	52	19	47	Bray (100)	82.7%satisfied

*abs* bioabsorbable, *GE* good to excellent, *OMAS* Olerud-Molander, *AOFS* American Orthopaedic Foot Ankle Society Hindfoot score, *N.A.* not available, *SMFA* Short Musculoskeletal Function Assessment, *GFA* Global Foot Ankle Score

Three studies compared the results of TightRope with syndesmotic screw fixation [9, 11, 59]. In the TightRope group two of these studies showed a higher AOFAS score (ten and six points increase, respectively), whereas one showed a three-point lower outcome on a modified score. A total of seven studies used a (non-modified) AOFAS score in the suture-button treatment group [9, 14, 34, 39, 42, 59]. The weighted average outcome of 133 patients in these six studies was 89.1 points, with an average study follow-up of 19 months. When comparing this outcome to literature on screw (metallic and absorbable) and bolt fixation, including only studies using the AOFAS score, six studies were identified with 253 patients and a weighted average score of 86.3 points, with an average study follow-up of 42 months [1, 9, 13, 16, 59, 63] (Table 3). Two studies reported a significant earlier return to work in the TightRope group [44, 59].

### Need for implant removal

Prominent suture-buttons or wound complications with a need for implant removal was reported in ten studies out of the 11 included on the treatment of syndesmotic disruption with a suture-button (Table 2). These studies treated 220 patients treated with a TightRope, of which 22 (10%) were removed at an average follow-up of 16 months, with a range of implant removal between zero and 25%. Twenty-four studies were identified within the last decade using metallic screws or bolts, which reported on the need for implant removal with an average follow-up of 24 months [5, 9, 11, 13, 16, 19, 20, 23, 25, 27, 28, 30, 32, 35, 37, 38, 45, 46, 51, 53, 56, 59, 62, 63]. In these studies a total of 866 patients were treated with a syndesmotic screw or bolt and in 449 cases the implant was removed (51.9%), usually prior to weight-bearing. The rate of implant removal ranged from 5.8 to 100%, depending on hospital protocol. In 12 studies syndesmotic screws were removed on a regular basis, e.g. in more than three-quarters of patients.

### Other complications

Besides the 11 studies treating 145 patients with a suture-button device, a few case reports were published on complications with the TightRope. Treon reported two syndesmotic widening (recurrent diastasis) and one synostosis [60]. A synostosis was also reported by Mason et al. [33]. Hohman et al. reported a distal tibial fracture two years after the placement of a suture-button [22]. These complications are however not specifically related to the use of a suture-button and similar complications occur also with the use of syndesmotic screws [2, 6, 7, 31, 48, 50].

## Discussion

The use of the TightRope system has increased rapidly over the last five years, and its use has recently been estimated to be 10% of applied techniques in syndesmotic disruptions in the United States [4]. The current review shows similar AOFAS outcome scores for the treatment with the TightRope system (average 89 points) and screw fixation (86 points), with a 2.2 times longer follow-up in the screw group.

Besides controversies on which diameter, placement height and number of cortices, the need for routine syndesmotic screw removal has frequently been subject to debate. This debate is fed by fear of screw breakage and expected limitations in range of motion. Even though the TightRope system was initially presented as a device that did not need removal, the rate of implant removal might be as high as 25%. In the current review it was 10% on average. Several authors have already made suggestions to lower the rate of implant irritation and subsequent removal [3, 21, 39]. In the literature on syndesmotic screw fixation this percentage is dependent on hospital protocol and is slightly over 50% on average. In a recent review the functional outcome did not differ in cases with retained or removed syndesmotic screws [47]. However, the level of evidence was dependent on five level-4, one level-2 and one level-1 studies, which indicates the need for additional studies comparing routine removal and removal on indication. On the other hand, the routine removal of syndesmotic screws has been associated with a high complication rate of over 20%, with both recurrent diastasis and wound infection following elective screw removal occurring in up to 10% [25, 48]. This, in combination with similar outcome scores, might suggest that syndesmotic screws only need removal on an indicative base.

Therefore to prove superiority of the TightRope system, it should be compared in a randomized controlled trial with three-cortical syndesmotic screws removed only on clinical indications. There are currently two studies ongoing or planned at ClinicalTrials.gov (Identifier: NCT01275924 and NCT01109303) comparing the TightRope system with syndesmotic screws.

Besides this new debate on which implant to use, new discussions have risen whether one or two suture-buttons should be used and in which configuration. Naqvi et al. placed a second TightRope in 26% and DeGroot et al. used more than one in 75% of their patients [14, 39]. Considering the long-term effects, the longest follow-up is currently approximately two years. In some studies, osteolysis, subsidence of the implant and enlargement of the tibial drill-hole at longer follow-up have been noted. Several authors therefore advise continued follow-up to monitor these effects and their possible influence on outcome [14, 22, 61].

A final point of consideration is the additional costs and subsequent cost-effectiveness of the TightRope system versus a syndesmotic screw. One abstract could be identified, which at this point does not answer these questions [43]. The additional costs of a syndesmotic screw removed in daycare surgery in the Netherlands are around 700 Euro, which is approximately the cost of two TightRope systems. There is currently no prospective research on the hospital and socio-economic cost-effectiveness of the TightRope system versus a syndesmotic screw, which takes the following items into consideration: additional surgery for implant removal, complications, number of follow-up clinic appointments, return to work and additional absence from work.

In conclusion, the use of a suture-button repair of a distal tibiofibular syndesmosis rupture leads to an earlier return to work, similar functional outcome as measured on the AOFAS score, and less frequent need for implant removal compared with the use of a syndesmotic screw. Further research is needed, with more uniformity in outcome reporting, on the long-term effects and cost-effectiveness of the treatment of acute distal tibiofibular syndesmotic disruption treated with a suture-button device.
